# Electrophysiological and Behavioral Responses of* Theocolax elegans* (Westwood) (Hymenoptera: Pteromalidae) to Cereal Grain Volatiles

**DOI:** 10.1155/2016/5460819

**Published:** 2016-01-21

**Authors:** Giacinto Salvatore Germinara, Antonio De Cristofaro, Giuseppe Rotundo

**Affiliations:** ^1^Department of the Sciences of Agriculture, Food and Environment, University of Foggia, Via Napoli 25, 71122 Foggia, Italy; ^2^Department of Agricultural, Environmental and Food Sciences, University of Molise, Via De Sanctis, 86100 Campobasso, Italy

## Abstract

Volatiles emitted by the host's food would be the first signals used by parasitoids in the host location process and are thought to play an important role in host habitat location. In this study, the olfactory responses of* Theocolax elegans* (Westwood), a Pteromalid wasp that parasitizes immature stages of stored-product insect pests developing inside cereal or leguminous grains, to volatiles emitted by healthy wheat grains, their hexane extracts, and different doses of three individual compounds previously identified in cereal grain odors were investigated in Y-tube olfactometer and Petri dish arena behavioral bioassays and electroantennogram recordings. In Y-tube olfactometer bioassays, odors from healthy wheat grains and their hexane extracts were attractive to both sexes of* T. elegans*. Moreover, hexane extracts elicited arresting effects in Petri dish arena. The three synthetic compounds valeraldehyde, maltol, and vanillin elicited dose-dependent responses in both male and female adult wasps confirming the capability of the peripheral olfactory systems to perceive cereal volatiles. In behavioral bioassays, different doses of vanillin were significantly attractive to both sexes.

## 1. Introduction

The use of natural enemies is considered to be an important component of integrated pest management of stored-grain insect pests [1], particularly in storage areas. Because the adult beneficial insects are external to the grain, they can easily be removed using normal grain-cleaning procedures [2].

In their search for hosts, food and mates parasitoids are guided by multisensory information including visual, vibrational, and olfactory signals [3–5] which are frequently used in an interactive manner [6–8].

The success of parasitic wasps in suppressing pest population depends on their ability to locate hosts; therefore, understanding the host location process is critical for the successful implementation of biological control programs. Host-seeking behavior in parasitoids has been divided into four phases, habitat location, host location, host recognition, and host acceptance [3, 9]. Orientation to host plant or host food volatiles in the absence of any host, host damage, and host derived materials have been demonstrated for a number of parasitic wasps [10–14], including some Pteromalidae species parasitizing stored-grain insect pests [15–17]. These volatiles, generally acting as long distance cues, would be the first signals used by parasitoids in the host location process and are thought to play an important role in host habitat location [18–20]. The identification of such synomones has a great practical interest since they could be used as parasitoid behavior modifying compounds to enhance their biological performances.

*Theocolax elegans* (Westwood) is a solitary ectoparasitoid of immature stages of economically important stored-product insect pests which develop as internal feeders of cereal and legume grains including* Rhyzopertha dominica* (Fabricius),* Sitophilus* spp.,* Sitotroga cerealella* (Olivier), and* Callosobruchus* spp. [21].* T. elegans* has been extensively studied as a biological control agent of some cereal pests. In large-scale experiments, the wasp was effective in reducing* Sitophilus zeamais* Motschulsky populations by up to 50% [22] and those of* R. dominica* by 50–99% depending on the temperature [23–25]. Moreover, augmentative release of* T. elegans* to control* R. dominica* resulted in a 61–92% reduction of the total number of insect fragments in wheat flour [2] indicating a positive impact on the quality of stored cereal products.

Preliminary behavioral bioassays suggested a preferential orientation of* T. elegans* adults to odors of healthy wheat grains [26] and the sensitivity of the antennal olfactory systems of both sexes towards a wide range of volatiles identified from various cereal grains [27–29] was shown by electroantennogram (EAG) recordings [30].

In the present study, the behavioral responses of* T. elegans* males and females to odors of uninfested wheat grains and their hexane extracts were carefully investigated using Y-tube olfactometer and Petri dish arena bioassays. Moreover, the biological activity of three individual volatiles (valeraldehyde, maltol, and vanillin) previously identified in the aroma of fresh cereal grains [27] and attractive to the two* T. elegans* hosts* Sitophilus oryzae* [31] and* S. granarius* [32] was investigated in a range of doses by electroantennographic and behavioural bioassays.

## 2. Material and Methods

### 2.1. Insects

A colony of* R. dominica* parasitized by* T. elegans* was collected from a cereal storehouse and set up in glass jars (15 cm diameter × 25 cm) half filled with uninfested wheat kernels and closed with fine metallic net (0.2 mm mesh). Colonies were maintained at 28 ± 2°C, 60 ± 5% relative humidity, and 12L : 12D photoperiod. During the following four months, wheat kernel samples (5 g) from the rearing jars were periodically transferred to transparent plastic containers (6 cm diameter × 8 cm) and held to check for the emergence of adult wasps. Emerging insects were collected daily and kept individually in glass vials (1.5 cm diameter × 5 cm) without food supply and in the absence of cereal odors for at least 12 h before the EAG and behavioral experiments. The sex of the insects tested was determined by observing their genitalia with a stereomicroscope.

### 2.2. Extract Preparation

Solvent extracts of wheat kernels (*Triticum durum* var. Simeto) were prepared by immersing wheat grains (40 g) in hexane (20 mL) for 36 hours at room temperature. Extracts from 3 grain samples were combined and concentrated to 10 mg grain equivalent/*μ*L using a gentle stream of nitrogen and stored at −20°C until being needed.

### 2.3. Chemicals

Valeraldehyde (1-pentanal), maltol (3-methoxy-2-methyl-4-pyrone), vanillin (3-methoxy-4-hydroxybenzaldehyde), and hexanal used as stimuli and hexane, mineral oil, acetone, and chloroform used as solvents were purchased from Sigma-Aldrich (Milan, Italy) and were 97–99% pure.

### 2.4. Y-Tube Olfactometer Bioassays

A glass Y-tube olfactometer (each arm 23 cm long at 75°C angle, stem 30 cm long × 3.0 cm i.d.) previously described [33] was used to examine the behavioral responses of* T. elegans* adults to odor stimuli. Each arm of the Y-tube was connected to a glass cylinder (9 cm long × 3.0 cm i.d.) as an odor source container. The apparatus was put into an observation chamber (90 × 75 × 40 cm) and illuminated from above by two 36-W cool white fluorescent lamps providing uniform lighting (2500 lux) inside the tube. A purified (activated charcoal) and humidified airflow maintained at 6 mL/min by a flowmeter was pumped through each arm.

In order to test the attractiveness of volatiles emitted by healthy wheat grains and their hexane extracts to* T. elegans* males and females two dual-choice experiments were performed: (1) wheat grains (25 g)* versus* clean air and (2) wheat grain hexane extracts* versus* control hexane. In this latter, the odor chamber contained a filter paper disk (1.0 cm^2^) loaded with 50 *μ*L of a 10 mg grain equivalent/*μ*L hexane extract, while the control chamber contained a filter paper disk loaded with 50 *μ*L of hexane. Both disks were suspended in the center of the cross section. In third set of experiments the behavioral responses of the adult wasps to different doses of vanillin were evaluated. The test stimulus was a filter paper disk loaded with 10 *μ*L of 5 *μ*g/*μ*L vanillin hexane solution, while a similar filter paper disk loaded with 10 *μ*L of hexane was used as control.

Individual* T. elegans* adults were released at the open end of the stem. Each experiment lasted for 5 min. A choice was recorded when the insect moved 3 cm up an arm of the Y-tube crossing the decision line (marked on both arms) and remained beyond that line for more than 30 sec. Insects that did not make a decision within 5 min were considered as no response and discarded.

After 5 individuals had been tested, the olfactometer was cleaned with acetone and dried (200°C for 30 min) and treatments between arms were switched to avoid position bias. For each test stimulus at least 20 male and female wasps were tested. A *χ* ^2^ test was employed to determine the significance of differences between the number of wasps choosing the treatment or control arm of the olfactometer.

### 2.5. Electroantennography (EAG)

The electrophysiological response of male and female* T. elegans* antennae to increasing concentrations of synthetic valeraldehyde, maltol, and vanillin was measured by the EAG technique used in previous studies [30, 34]. Antennae were excised from 1- to 2-day-old insects. The base of the antenna was put into a glass pipette filled with Kaissling saline [35] which served as the neutral electrode. The tip of the antenna was put in contact with the end of a similar pipette (0.1–0.2 mm diameter) which provided the active electrode.

AgCl-coated silver wires were used to maintain the electrical continuity between the antennal preparation and an AC/DC UN-6 amplifier in DC mode connected to a PC equipped with the EAG 2.0 program (Syntech Laboratories, Hilversum, Netherlands). Stimuli were serial solutions (0.0125, 0.025, 0.05, 0.1, 0.15, and 0.2 mg/*μ*L) of valeraldehyde, maltol, and vanillin, respectively, dissolved in mineral oil, acetone, and chloroform (Sigma-Aldrich) to achieve a satisfactory solution. Just before the experiment, 20 *μ*L of each test solution was adsorbed onto a filter paper strip (1 cm^2^, Whatman number 1) placed in a Pasteur pipette (15 cm long), which served as an odor cartridge. After complete evaporation of the corresponding solvent, stimuli were blown by a disposable syringe into a constant stream of charcoal-filtered humidified air (500 mL/min) flowing in a stainless steel delivery tube (1 cm diameter) with the outlet positioned at approximately 1 cm from the antenna. Over 1 s, 2.5 cm^3^ of vapor from an odor cartridge was added. Stimuli were applied in ascending dose [36] and randomly sequenced for each dose. Control (20 *μ*L of mineral oil) and standard (20 *μ*L of 50 *μ*g/*μ*L hexanal mineral oil solution) stimuli were applied at the beginning of the experiment and after each group of 3 test odors. Intervals between stimuli were 30 s. For each compound, EAG responses were recorded from 5 antennae of different insects of each sex.

The amplitude (mV) of the EAG response to each test stimulus was adjusted to compensate for solvent and/or mechanosensory artifacts by subtracting the mean EAG response of the two nearest mineral oil controls [37]. To compensate for the decrease of the antennal responsiveness during the experiment, the resulting EAG amplitude was corrected according to the reduction of the EAG response to the standard stimulus [38]. The activation threshold of dose-response curves was considered to be the lowest dose at which the lower limit of the standard error of the mean response was greater than the upper limit of the standard error for the lowest dilution tested [39]; saturation level was taken as the lowest dose at which the mean response was equal to or less than the previous dose [30]. For each dose tested, the mean EAG responses of each sex to the three compounds were subjected to analysis of variance (ANOVA), Levene's test of homogeneity of variance and ranked according to Tukey's HSD test. Male and female EAG responses to a set test dose of each compound were compared using Student's* t*-test.

### 2.6. Petri Dish Arena Bioassays

The behavioral response of* T. elegans* males and females to the hexane wheat extract (10 mg grain equivalent/*μ*L) and valeraldehyde, maltol, and vanillin solutions (0.01, 0.1, 1.0, and 5.0 *μ*g/*μ*L) in hexane [31] was assessed in an arena consisting of a glass Petri dish (15 cm diameter × 2 cm height).

The base of the dish was divided into four sectors. An aliquot of the test stimulus (50 *μ*L of hexane wheat extract or 10 *μ*L of a compound solution) was adsorbed onto a filter paper disk (1 cm^2^, Whatman number 1) and placed in one sector after solvent evaporation (2 min). The remaining sectors contained similar filter paper disks treated with equal volumes of hexane used as controls. Treatment and control stimuli were allowed to diffuse into the arena for 3 min before the experiment started. The Petri dish was placed on the bottom of a white box (90 cm × 75 cm × 40 cm) and illuminated from above resulting in 400 lux on the dish.

An insect was released at the center of the Petri dish and its allocation in the four sectors was recorded for 5 min. After 5 individuals were tested, the olfactometer was cleaned with acetone and dried (200°C for 30 min). The position of the odor sample in the Petri dish was changed routinely to avoid position bias. For each treatment, 15 males and females used once were tested. The time spent by insects in the four sectors was analyzed by Friedman two-way ANOVA by ranks and in the case of significance (*P* < 0.05) the Wilcoxon signed ranks test was used for separation of means. Analyses were performed with SPSS (Statistical Package for the Social Sciences) version 10.0.7 for Windows (SPSS Inc., Chicago, IL).

## 3. Results

### 3.1. Behavioral Response to Wheat Grain Volatiles

**Figure 1:**
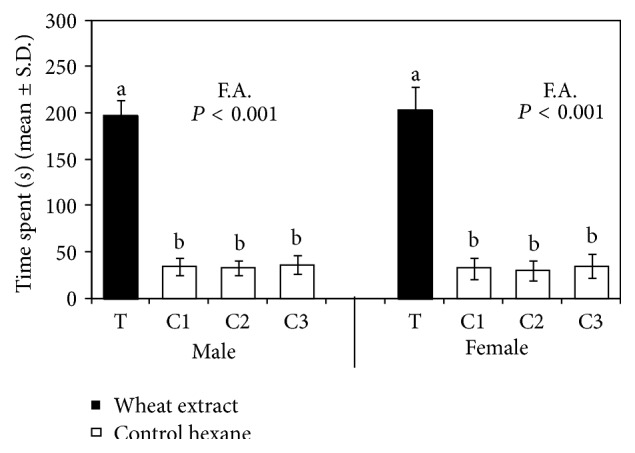
Mean allocation time of* T. elegans* males (*N* = 20) and females (*N* = 20) in the sectors of a Petri dish provided with 50 *μ*L of hexane extract of wheat grains (500 mg grain equivalent) (T) or with 50 *μ*L of hexane (C1, C2, and C3). Means were submitted to Friedman's two-way ANOVA (F.A.). For each sex, values with different letters are significantly different according to Wilcoxon test (*P* ≤ 0.001).

In Y-tube behavioral tests, the percentage of adult wasps responding to stimuli tested was generally high (Table 1). Odors from wheat grains elicited a significant attraction in both male and female wasps. When insects were presented with wheat grain extract* versus* control hexane a significant preference for wheat extract was exhibited by both males and females. The mean allocation time of* T. elegans* males and females in the sector of Petri dish provided with wheat extract was significantly higher (Friedman test, *P* < 0.001; Wilcoxon test, *P* < 0.001) than those in related controls (Figure 1).

### 3.2. EAG

**Figure 2:**
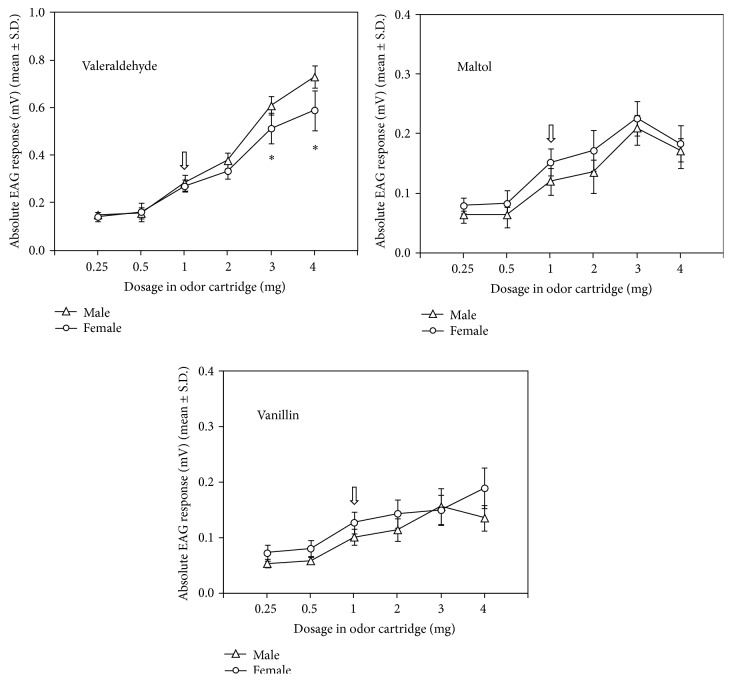
Electroantennogram dose-response profiles of* T. elegans* males and females (*n* = 5) to valeraldehyde, maltol, and vanillin. Arrows indicate the activation thresholds. Asterisks indicate significant differences between male and female EAG responses (Student's* t*-test, *P* = 0.01).

The sensitivity of male and female* T. elegans* antennae toward increasing concentrations of valeraldehyde, maltol, and vanillin was reported in Figure 2. All compounds elicited EAG dose-dependent responses with action thresholds at the 1 mg dose in both sexes. The amplitude of the EAG response of both sexes to maltol and that of males to vanillin decreased from 3 to 4 mg doses indicating saturation of receptors at the lowest dose.

For each dose tested, the mean EAG response to valeraldehyde was significantly higher than those to vanillin and maltol in both males (*F* = 60.10–593.27, d.f. = 2, *P* < 0.001) (*P* < 0.05, Tukey-HSD test) and females (*F* = 27.94–95.84, d.f. = 2, *P* < 0.001) (*P* < 0.05, Tukey-HSD test). In both sexes, no significant differences were found between the EAG responses to maltol and vanillin at different doses. Male EAG responses to valeraldehyde were significantly higher (*P* < 0.05,* t*-test) than those of females at the 3 and 4 mg doses. Male and female EAG responses to each dose of maltol and vanillin did not differ significantly (*P* > 0.05,* t*-test) (Figure 2).

### 3.3. Behavioral Response to Individual Grain Volatiles

**Figure 3:**
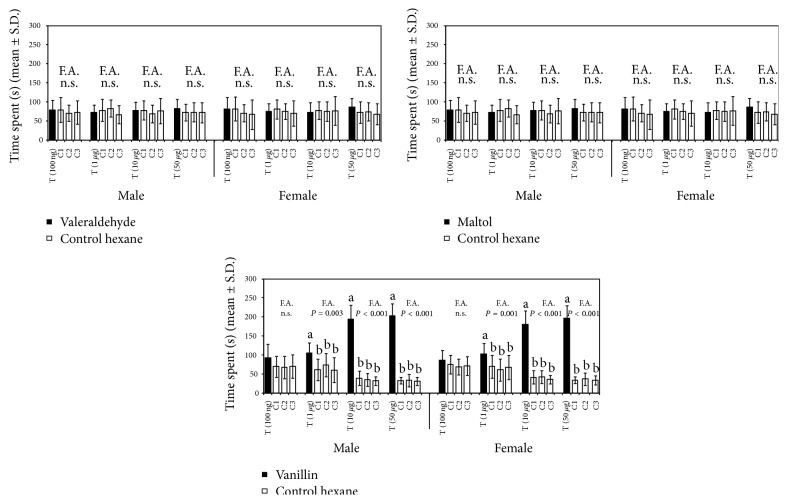
Mean allocation time of* T. elegans* males (*N* = 15) and females (*N* = 15) in the sectors of a Petri dish provided with increasing doses of synthetic compounds (T) or with 10 *μ*L of hexane (C1, C2, and C3). For each dose tested, means were submitted to Friedman's two-way ANOVA (F.A.) followed by Wilcoxon test in case of significance (*P* < 0.05). Values with different letters are significantly different according to Wilcoxon test (*P* ≤ 0.01).

In the range of dose tested, the mean allocation times of* T. elegans* males and females in the sector of Petri dish provided with maltol or valeraldehyde were not significantly different (Friedman test, *P* > 0.05) from those of related controls (Figure 3). A similar result was observed on applying the 100 ng dose of vanillin. Using the 1 *μ*g dose of this compound the mean allocation time in the treatment sector was significantly higher than those in controls in both males (Wilcoxon test, *P* = 0.003) and females (Wilcoxon test, *P* = 0.001) (Figure 3). At the 10 and 50 *μ*g doses, the mean time spent by male and female wasps in the sector with vanillin was significantly higher (Wilcoxon test, *P* < 0.001) than those in controls and higher than those recorded at the 1 *μ*g dose (Figure 3). At the 50 *μ*g dose, vanillin elicited a significant attraction of male (*χ* ^2^ = 7.20, d.f. = 1, *P* = 0.007) and female (*χ* ^2^ = 9.80, d.f. = 1, *P* = 0.002) wasps in Y-tube olfactometer bioassays.

## 4. Discussion

*T. elegans* males and females preferred volatile compounds emitted by healthy wheat grains when offered as an alternative to an empty control in the absence of visual stimuli in a Y-tube olfactometer. Volatile compounds present in hexane extracts of healthy wheat grains were preferred to control hexane in the same apparatus and elicited an arrestant effect in Petri dish arena, thus suggesting the presence of behaviorally active compounds acting as attractants at long distance and arrestants at short range. This implies that both sexes of* T. elegans* are able to detect host substrate on the basis of chemical cues and confirm their capability to perceive cereal grain odors [30]. Plant volatiles are used by female parasitic wasps to locate the habitat of host insects, while male wasps use the same chemical cues to locate the females for mating [40], probably in combination with a sex pheromone.

When, for instance, host population has declined or the host have dispersed from the emergence site, parasitoids may search for a suitable environment with adequate food sources [3, 41]. According to our results, volatiles released by cereal grains more likely drive* T. elegans* adults in the search for a suitable host habitat outside and inside cereal storage.

A possible explanation for the attraction of* T. elegans* adults to cues of healthy grains may be that being hosts concealed inside grains, seed odors probably act as a substitute in situations in which other host related cues, that is, host feces outside the seeds or specific volatiles released by herbivory-damaged grains, are not present to indicate host presence [42]. In this situation, the ability to respond to uninfested grains may serve to prolong parasitoid foraging activity [43, 44] and to exploit a wider range of hosts [42] within the habitat. Alternatively, the blend of volatiles released by healthy seeds may be very similar to that produced by host-damaged seeds causing the parasitoid to respond anyway, at least at long distance. To test this hypothesis, pairwise comparisons of infested and uninfested grains in olfactometer bioassays in combination with chemical analyses of volatile compounds are needed.

The importance of healthy seed volatiles to Pteromalid wasps parasitizing immature stages of internal feeders of cereal or legume seeds was highlighted by previous studies [15–17]. For instance, in pairwise comparisons of attractant stimuli in behavioral bioassays mated females of* Pteromalus cerealellae* (Ashmead) showed preference for the extract of uninfested cowpea seeds compared to larval frass extract [45]. In* Lariophagus distinguendus* (Förster) host feces alone were not attractive to female wasps while healthy grains were attractive even if less than a combination of host feces and herbivore damaged grains [42] indicating positive short range interactions between host and host food cues.

EAG studies showed that* T. elegans* males and females are able to detect the three fresh grain volatiles used in this study. This is in accordance with results of behavioral bioassays and provides further evidences that hosts' food volatiles are used by both male and female parasitic wasps.

For both sexes, different EAG dose-response curves to selected compounds were recorded with valeraldehyde being the most active at all doses tested. This could reflect differences in the number of specific antennal receptors involved in the perception of different cereal volatiles, more than differences in volatility of compounds, and indicate differential selectivity and sensitivity of the peripheral olfactory systems for host habitat odors [40, 46, 47]. Differences in the number of olfactory receptor neurons tuned to different compounds could also explain sexually dimorphic responses observed to high doses of valeraldehyde probably as a result of different roles played by the same plant volatile in the ecology of males and females.

In the dose range from 1 to 50 *μ*g, a significant arresting effect of vanillin to* T. elegans* males and females was observed in Petri dish arena bioassays whereas, in the same experimental conditions, maltol and valeraldehyde did not influence the parasitoid behavior. Moreover vanillin was preferred to control hexane in Y-tube olfactometer bioassays. It is worth noting that the behavioral responses elicited by vanillin were comparable to that induced by wheat grain hexane extracts in both apparatuses and even comparable to that of wheat grains in Y-tube olfactometer. Despite the strong attractant activity of vanillin to* T. elegans* adults, the EAG responses evoked by this compound in the range of doses tested showed only moderate amplitudes in both sexes. This is not surprising because a weak correlation between EAG and behavioral activity can occur and it is likely that a few sensilla specifically tuned to an important chemical cue would enhance its detection in the complex blend of plant-based host related volatile compounds [30]. Vanillin was already found to be attractive to* S. oryzae* [31] and* S. granarius* [32]. The sensitivity of a parasitoid and its host to the same volatile compounds emitted by host plants was also observed in other tritrophic systems [40, 48–51].

## 5. Conclusions

Results of behavioral bioassays demonstrated strong attractant activity of odors emitted by healthy wheat grains to both sexes of* T. elegans*. Electroantennogram recordings in response to three fresh cereal grain volatiles confirmed the capability of the peripheral olfactory systems of male and female wasps to detect chemical cues in a range of doses. Finally, in behavioral bioassays with the same compounds, vanillin was shown to be an effective attractant for both sexes of* T. elegans*. To the best of our knowledge, vanillin is the first synomone of a member of the* Theocolax* genus. This compound could be useful for detecting* T. elegans* and manipulating the parasitoid behavior to ensure suitable population levels in the environments where its hosts are present.

## Figures and Tables

**Table 1:** Behavioral response of *Theocolax elegans* adults in a Y-tube olfactometer to different sources of wheat grain odors.

Odor sources	Male				Female			
	*N* ^a^	Treated^b^	*χ* ^2^	*P* value^c^	*N* ^a^	Treated^b^	*χ* ^2^	*P* value
Wheat grains versus control air	20 (20)	18	12.8	<0.001	20 (20)	19	16.2	<0.001
Wheat grain extract versus control hexane	21 (20)	16	7.2	0.007	21 (20)	18	12.8	<0.001

^a^Total sample size (*N*: number of individuals that made a choice in parentheses).

^b^Number of individuals (of those that made a choice) that chose the treated arm first.

^c^d.f. = 1 for all *χ* ^2^ reported.
